# The Venereal Disease Inquiry

**Published:** 1923-07

**Authors:** 


### THE VENEREAL DISEASE INQUIRY.

ALTHOUGH the original terms of reference, under which the Committee of Inquiry on Venereal Disease have just published their report (H.M. Stationery Office, 3d. net), included medical measures only, it is well that opinions are also expressed on certain other conditions which directly affect the community. In this way a much more valuable and conclusive report has become available. Great stress is quite rightly laid upon the need for public extension of knowledge as to the nature of the diseases and their remoter consequences. As of no less importance was mentioned the removal of conditions of life which foster the disease. Thereafter the report turns to directly medical matters and discusses the problem from the points of view of preventing disease in those exposed to infection and curing those already infected. With regard to the first the somewhat reserved comment is made that, in a community where there has been efficient instruction and where there was such a condition as arises from the control and influence present in a military body under efficient command, substantial results may be expected from prophylactic measures.

### TREATMENT IN RURAL DISTRICTS.

It is thought, however, that there is no justification for putting obstacles in the way of individuals who desire to procure disinfectants, and that the law should be altered to permit qualified chemists to sell *ad hoc* disinfectants in approved form and with approved instructions. Commercial advertisement should be prohibited, however, and money spent on a general disinfecting system is not advised. The existing clinics and their work are strongly commended, and the question is opened of extending systematic treatment to the rural districts by constituting a panel of practitioners through whom first-class treatment could be obtained. Notification is wisely disapproved. The more complete treatment of these cases in Poor Law hospitals is urged.

### ONE NEW SUGGESTION.

The only organised ablution centres which are considered useful are such as might be organised in connection with seaport towns and docks, or in special areas where they would be undeniably useful. Otherwise no entirely new suggestions are made. So much controversy has lately arisen upon the subjects here dealt with that it is highly satisfactory to see the whole matter so clearly and moderately put forward. We trust the care the Committee have put into their work will be rewarded by future unanimity between the various bodies coping with the problem, both directly and indirectly. However good the ultimate objective may be, results cannot be the best while constant differences of opinion occur among those working in the cause.

